# Mapping Variation in Breast Cancer Screening: Where to Intervene?

**DOI:** 10.3390/ijerph16132274

**Published:** 2019-06-27

**Authors:** Cindy M. Padilla, François Painblanc, Patricia Soler-Michel, Veronica M. Vieira

**Affiliations:** 1 Université Rennes, EHESP, REPERES (Recherche en pharmaco-épidémiologie et recours aux soins)—EA 7449 Rennes, France; 2 Centre régional de coordination des dépistages des cancers Auvergne Rhône Alpes, 5 bis, rue Cléberg, 69322 Lyon CEDEX 05, France; 3 Program in Public Health, University of California, Irvine, CA 92697, USA

**Keywords:** mammography screening, opportunistic screening, breast cancer, spatial variation, mapping, socioeconomic inequalities

## Abstract

Small geographic areas with lower mammography screening participation rates may reflect gaps in screening efforts. Our objective was to use spatial analyses to understand disparities in mammography screening use and to identify factors to increase its uptake in areas that need it in Lyon metropolitan area, France. Data for screened women between the ages of 50 and 74 were analyzed. Census blocks of screened and non screened women were extracted from the mammography screening programme 2015–2016 dataset. We used spatial regression models, within a generalized additive framework to determine clusters of census blocks with significantly higher prevalence of non-participation of mammography screening. Smoothed risk maps were crude and adjusted on the following covariates: deprivation index and opportunistic screening. Among 178,002 women aged 50 to 74, 49.9% received mammography screening. As hypothesized, women living in highly deprived census blocks had lower participation rates compared to less deprived blocks, 45.2% vs. 51.4% *p* < 0.001. Spatial analyses identified four clusters, one located in an urban area and three in suburban areas. Moreover, depending on the location of the cluster, the influence came from different variables. Knowing the impact of site-specific risk factors seems to be important for implementing an appropriate prevention intervention.

## 1. Introduction

With 58,968 new cases in 2017, breast cancer is the most common cancer observed in women in France, as well as the European Union [1]. In 2017, breast cancer ranked first in mortality, with more than 12,000 deaths, although it was closely followed by lung and colorectal cancer [1]. Mammography screening tests are important tools in combating cancer-related morbidity and mortality. Early diagnosis allows more effective treatment in most cases of cancer, with a positive impact on the prognosis of the disease [2].

Despite organized efforts to promote breast cancer screening, large disparities in rates of mammography persist in a Canadian [3,4,5], American [6,7,8,9,10,11] and European setting [12]. In France, the National Mammography Screening Programme (NMSP) has been generalized to the whole country since 2004, after pilot experiences. Women aged 50–74 years are invited by a letter, every two years, to receive a free mammogram. After 6 months, a reminder is sent to those who failed to attend. Following the recommendations of the National Authority for Health, the examination includes two steps: a clinical examination by the first reader and immediate assessment in the event of a positive result, with a centralized double reading of all negative screens and recall in the case of a positive after double reading. Participation in this programme remains low (around 52% in 2016–2017, according to the French Institute for Public health Surveillance), below the acceptable participation rate of 70% recommended to keep the programme cost-effective [13]. 

Characteristics of communities and areas in which women reside play an important role in breast cancer screening [14,15,16,17]. Previous studies have demonstrated socioeconomic inequalities in mammography screening with women located in a low-income neighborhood less likely to get screened [3,4,18,19]. They suggest that higher median income and higher educational level within an area are associated with higher levels of mammography screening uptake [18]. Other studies have demonstrated the same association using a global indicator of deprivation [19,20,21]. Moreover, geography, attitudes and beliefs about screening services may predispose women to screening utilization. A study demonstrated that women in urban areas with a higher level of socioeconomic status were more exposed to opportunistic screening than in suburban or rural areas [22]. Opportunistic screening does not offer the same level of quality screening as the NMSP, which is accredited and includes double readings [23]. In Luxembourg, a policy of organising the screening process by reimbursing only those mammograms done within the context of the organised screening programme was implemented to drastically reduce opportunistic screening [24]. 

The use of a Geographic Information System (GIS) to visually depict cancer-relevant health data is well documented in the cancer literature [25,26,27,28]. However, only a few studies have employed spatial mapping to determine if prevalence of mammography screening are equally distributed throughout the study setting [3,10,11], and no such study has been conducted in France. Spatial analyses have the potential to inform public health scientists, actors from NMSP and policymakers about areas of need in relation to service provision and to aid in the development of the most appropriate interventions to increase use of preventive services [15,17]. Mapping cancer screening data is a powerful tool for local cancer associations and academics working together to increase access to services.

In this paper, we conducted spatial analyses to detect and understand the geographic disparities in the participation of mammography screening between 2015 and 2016 in Lyon metropolitan area (MA). The two main objectives of the study are: (i) to identify clusters of census blocks with significantly higher prevalence of non-participation of mammography screening, (ii) to present contextual characteristics of the different clusters to determine their regional attributes.

## 2. Date and Methods 

### 1.1. Data and Geographic Indicator Description

#### 1.1.1. Location

Lyon MA is the third most important metropolitan area in France. It is composed of 59 municipalities and 510 census blocks in the region of Auvergne-Rhône-Alpes in the Eastern region of France, with a total population of 1,381,349 inhabitants in an area of 534 km^2^ in 2016. The city of Lyon contains 37% of the regional population. Although predominantly urban, 40% of the metropolitan area is composed of natural and agricultural areas. This area is of particular interest for studying geographic disparities in mammography screening because it contains both rural and urban areas and their urban landscapes are contrasted in terms of certain significant demographic and socioeconomic characteristics. Moreover, actors of the regional mammography screening programme have realized previous interventional studies in deprived census blocks of the region [20]. Unfortunately, the global mammography screening participation rate in the region of Auvergne-Rhône-Alpes has not decreased and social inequalities in cancer screening participation persist.

The geographical boundaries for the census blocks were obtained from the National Geographic Institute. A census block is the smallest geographical unit for which French National Institute of Statistics and Economic Studies (INSEE) data are available and correspond to a small neighbourhood of approximately 2000 inhabitants. The geographic position of each census block is required as input for the spatial analyses, so we derived the position of the centroid (a measure for the geographical center point of a polygon) for each census block using a zonal geometry function in GIS. All GIS techniques and map layouts were performed using ArcMap v.10.5 (ESRI, Redlands, CA, USA).

#### 1.1.2. Dependant Variable

**Figure 1:**
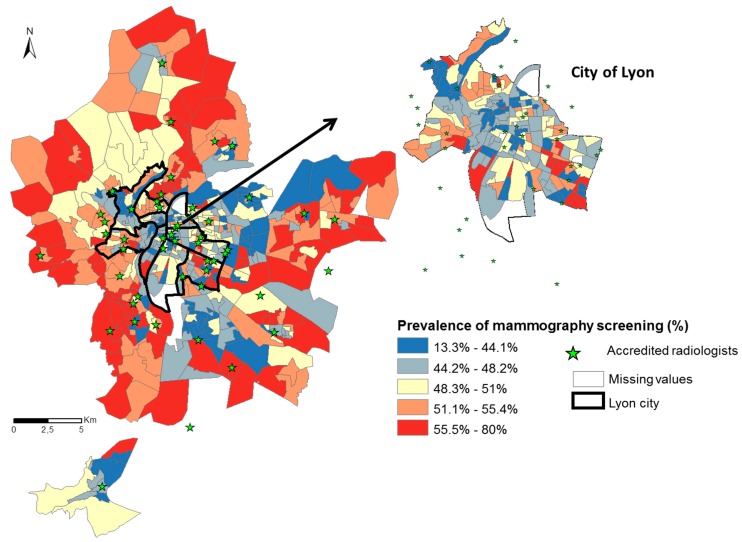
Spatial distribution of the prevalence of mammography screening and the location of accredited radiologists in 2015–2016, Lyon MA.

To identify census blocks that potentially need interventions to increase the mammography screening uptake, we focused our study on the prevalence of non-participation of mammography screening by census blocks. Census blocks of screened and not-screened women were extracted from the regional mammography screening programme 2015–2016 dataset of the Auvergne Rhone Alpes region. Women resident in Lyon MA, aged 50 to 74 years in 2015 to 2016, were analyzed at the time of invitation to attend for mammography screening. Women received with their invitation letter a list of accredited radiologists to contact them. The locations of these radiologists are presented in Figure 1, with the prevalence of mammography screening by census blocks for 2015–2016 in Lyon MA.

#### 1.1.3. Contextual Variables

To take into account the opportunistic screening, the proportion of women aged 50 to 74 who arranged for a mammography unassociated with the national screening programme (i.e., using a radiologist not listed in the invitation letter) were analyzed. Data were obtained according to the mammography reimbursement data from the general medical care. 

**Figure 2:**
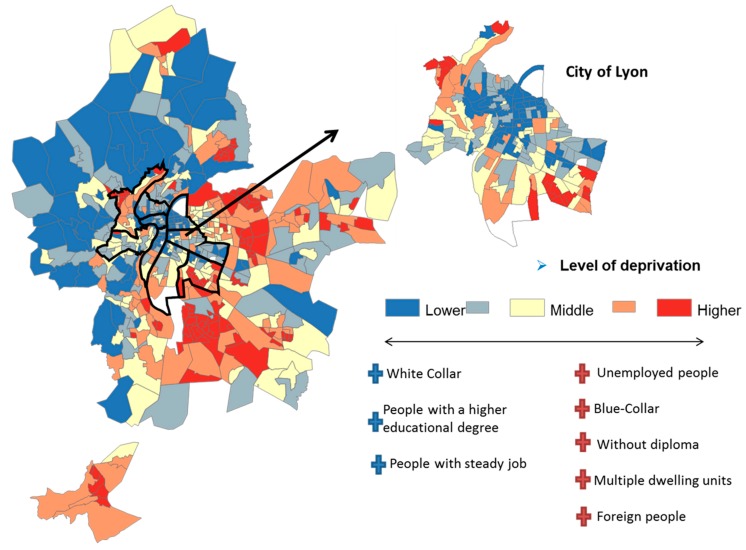
Spatial distribution of the deprivation index in quintiles in Lyon MA.

Socioeconomic characteristics refer to the social and economic factors that could influence behaviors within the society [29]. An index of deprivation was calculated at the census block level. In short, the socioeconomic data were obtained from the 2014 national census and provided counts of population, households or residences at the census block level classified by social, economic and demographic characteristics. Census block-level variables in the followings domains: employment, single parent family, education, occupation, immigration status, proportion of social housing, were constructed at the census block level according to INSEE’s definitions. Principal components analysis was used to synthesize information from these data. To construct a single numeric index for all of the blocks, we maximized the inertia of the first component by deleting all of the variables only weakly correlated with it and the variables with a contribution lower than the average. This indicator, developed by Lalloue et al. in 2013 [30], has been defined and analyzed in previous studies in order to analyze environmental and health inequalities [31]. Figure 2 presents the spatial distribution of the deprivation index, with red areas representing the most deprived census blocks and blue areas the lowest deprived census blocks.

Lyon MA is mostly an urban metropolitan area so instead of using an urban–rural indicator, we selected the following variables more appropriate to the study setting: the personal and professional mobility of each census block (different way to do home to work transport, stability or frequent relocation of home), housing description, and car and home owners. Data are available and come from the INSEE national census of 2014.

#### 1.1.4. Spatial Analysis

Spatial autocorrelation was tested for and a greater likelihood was found with similar mammography screening rates in adjacent census blocks than among census blocks further away. The objective was to estimate local correlations for each census block to identify clusters of census blocks with significantly higher prevalence of non-participation of mammography screening. Cases were women ages 50–74 who did not participate in the national mammography screening program. The generalized additive model (GAM) was used with a Poisson distribution and an offset for the number of women invited by census blocks.

We modelled location, a potential proxy measure of unknown exposure or uncontrolled risk factors, using a smooth (S) of longitude (X) and latitude (Y) with a Poisson link function.

*log*[*p*(*x*1, *x*2)] = *S*(*x*1, *x*2) +offset (pop) + *γz* (4) (1)

where the left-hand side is the logarithm of the risk of non-participation at the census block’s centroid (X,Y), according to the size of the population, while adjusting for confounding variables, γz, which are modelled parametrically [32]. The log of the relative risk at location (x1, x2) is modelled as it would be with a Poisson regression on the covariates if there was no smooth term.

A locally weighted regression smoother (LOESS) was used in the analyses; because of the non-homogeneity of population densities, disparities exist between census block according to the number of women invited to participate. The LOESS predicts the log odds by fitting a regression to data points closest to the prediction point and by weighting the data points with a tri-cube function of their distance from the prediction point [33]. The optimal span size (percentage of data points used for smoothing) was determined by minimizing Akaike’s information criterion, which is a trade-off between bias and variance. Small span sizes produce bumpier surfaces and larger span sizes produce smoother surfaces. As the span size increases, the amount of bias in the fit increases and the variance decreases.

GAMs also provide a framework for testing hypotheses. The null hypothesis of the global deviance test is that spatial distribution of the map is homogeneous, the mammography screening rates does not depend on location using the difference of the deviances of the complete model (Equation 1) and the reduced model omitting the smoothing term. The R software provides an approximate p-value for this statistic assuming a chi square distribution. To test the null hypothesis of the local test, we calculated the p-value using a permutation test [34]. We randomly reassigned the coordinates of the census blocks while keeping the case counts, population, and covariates fixed. From the null permutation we sampled the distribution 999 times in addition to the original model. For each permutation, we ran the GAM using the optimal span of the original data and computed the deviance statistic. We divided the rank of the observed value by 1000 to obtain the permutation p-value. If the deviance global statistic indicated that location was significant at the 0.05 level, we then identified areas with significantly increased risk. We identified areas of significantly elevated risk (“hot spots”), as all census blocks ranking in the upper 2.5% of the census block distributions, and denoted these areas with a black contour line in the resulting maps [32,35]. Spatial analyses were conducted in the R Package (version 3.0.1) using the “MapGam” package for the GAM approach.

#### 1.1.5. Analytical Strategy

In order to identify small geographic areas that may reflect gaps in screening efforts. We first performed a spatial analysis using the crude model to determine the unadjusted geographic variation in the prevalence of non-participation in the NMSP. Then covariates with a geographic component were added to the adjusted model and the maps were visually inspected to see if the variable changed the appearance of the map surface, decreasing or not the size of the cluster. Three adjusted analyses were conducted: (1) the deprivation index alone, (2) the deprivation index and proportion of women with non-programme related screenings and (3) the deprivation index and the proportion of women in the census block aged 50 or more as a proxy of social cohesion. Census blocks without any women invited to participate to organized breast cancer screening (for example, an industrial census block or a park) were excluded from the analysis (n = 13, 2.5%).

## 3. Results

### 3.1. Geographic Disparities in Mammography Screening 

During the years 2015–2016, the prevalence of mammography screening in Lyon MA was 49.9% [min = 13.3%, max = 80%]. In terms of deprivation level, the prevalence was lower for the highly deprived census blocks with 45.2% ± 7.9 and highest for the middle and favored census blocks with 51.4% ± 6.1 and 51.2% ± 6.1, respectively (*p* < 0.001, Figure 1). Looking at the location, favored census blocks in the western part of Lyon MA had the highest participation rate (53.7% ± 6.2). These census blocks are characterized by more rural than urban residential areas with a higher percentage of: homeowner, at least one car per household, not moved for more than 10 years and home to work transport by car (Figure 2). In comparison, the favored census blocks in the city center of Lyon (46.6% ± 7.5) had the lowest prevalence of mammography (*p* value < 0.001). The census blocks in the city center are characterized by highly-urban areas with a higher percentage of: non-owner households, not moved for less than two years, household of small surfaces and home to work transport by public transport (Figure 2). 

### 3.2. Focus on Areas of Non-Participation of Mammography Screening

**Figure 3:**
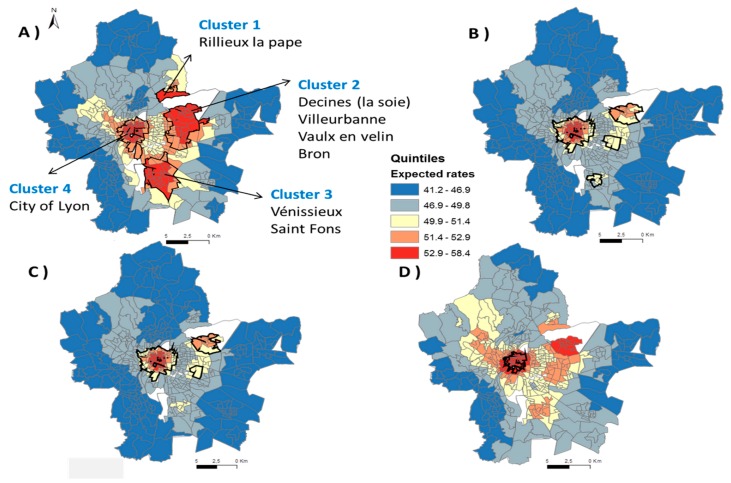
Estimated prevalence of non-participation of mammography screening by the GAMs in the Lyon MA for the crude model (**A**), according to (**B**) the deprivation index, (**C**) SES and individual screening and (**D**) SES and women aged 50 years and more. Solid lines identify areas with significantly lower participation rates of mammography screening compared to the rest of the study settings (hotspots).

Figure 3 shows clusters identified in the unadjusted and adjusted analyses for the expected risk of non-participation in the NMSP at the census block level, and Table 1 describes the contextual characteristics of the census blocks within each cluster. The results of the global hypothesis tests were *p* < 0.001 for all analyses, suggesting statistically significant clusters of census blocks with higher non-participation rates compared to the rest of the study area. Cluster 1 is composed of two census blocks and is located in the rural suburb of Lyon MA in Rillieux la pape. Cluster 2 and Cluster 3 are located in the close suburban area and are composed, in majority, by highly deprived census blocks (deprivation index of 78.4% and 82.6%, respectively). Cluster 2 is located in Villeurbanne, Vaulx en Velin, and Bron, and Cluster 3 is located in Venissieux and Saint Fons. Cluster 4 is located in the urban city center of Lyon and among all the census blocks included in this cluster, 71.9% are in low deprivation census blocks. 

After adjustment for the deprivation index, Cluster 1 was no longer statistically significant and the expected non-participation rates were reduced (Figure 3B). This strongly suggests that socioeconomic status explains the spatial variation of non-participation in the NMSP in Rillieux la pape. After adjustment for the deprivation index alone, the higher non-participation in the NMSP in Cluster 2 was reduced and the cluster size decreased by 51% of the census in Villeurbanne and Bron. When a higher level of deprivation and a higher proportion of the women aged 50 or more were both considered, the risk of non-participation once again increased but was no longer statistically significant for Vaulx en Velin city (Figure 3D).

Cluster 3, located in Venissieux and Saint Fons, decreased by 78% after adjustment for the deprivation index alone compared to the crude map (Figure 3B compared with Figure 3A). When we adjusted for the deprivation index and the proportion of opportunistic screening unrelated to the NMSP, Figure 3C shows there are no longer any census blocks with statistically significant non-participation in the NMSP within Cluster 3. This suggests that in Saint Fons and Venissieux, high deprivation explains the majority of the spatial variation in high non-participation in the NMSP in this area.

Non-participation in the NMSP remained high in the city center of Lyon (Cluster 4), as, after adjusting for deprivation, we found similar maps (data not shown), suggesting that the level of deprivation in the city of Lyon does not explain the spatial variation found for the participation of opportunistic screening. When the effect of the women aged 50 or more was taken into account in the model, the magnitude of the lower participation of mammography screening decreased compared to the crude map. In the city center, the density of the population is high and the population is active and younger. However, this variable does not entirely explain the spatial variability, and some other unknown variables are needed to explain this spatial variability in the city center of Lyon.

## 4. Discussion

The spatial analyses presented in this paper provide a general overview of where non-participation in the NMSP is higher within Lyon MA between 2015 and 2016. We identified four clusters of census blocks with higher non-participation of mammography screening located in different regions of the study setting (suburban, urban area and the smallest one in a rural area). Our results suggest that participation in the NMSP varied significantly across census blocks with inequitable deprivation levels. Nevertheless the results indicate that the deprivation of the area of residence should not be taken into account alone but combined with other factors, with the aim of removing areas of low participation of the mammography screening programme. These combinations of factors are specific to each cluster and should not be generalized throughout the Lyon MA.

We demonstrated that except for the city center of Lyon, the socioeconomic deprivation explained a large portion of variation in the low participation of mammography screening, confirming previous research that reported a lower participation rate in the highly deprived area [18]. Similar results have been found in France using other deprivation indices [19,20,34,35], or using individual socioeconomic factors [36,37]. The majority of the highly deprived census blocks are located in the close suburban area where two clusters were identified.

The results suggest that the location of the clusters is important. We found one significant cluster in an urban and active area of the city of Lyon, and a small cluster (2 census blocks) in a residential and rural part of the study setting; both of these clusters include low deprivation census blocks of Lyon MA. Contrary to previous studies, the participation rates in the NMSP is highest in the more rural and residential areas and lower in the dynamic areas of the city center of Lyon. Other studies demonstrated that rural areas are associated with women who never had a mammography and with lower participation of mammography screening according to the further distance to the mammography services [5,12,20,38,39]. A study by Maheswaran and Leung (2006) showed no differences between rural and urban areas [40].

We also demonstrated that some factors could modify the relation between deprivation and participation of mammography screening. The spatial analyses suggested that in Cluster 2, more precisely the city of Vaulx en Velin, after accounting for deprivation and a lower proportion of women aged 50 years or more (11%), the expected risk of non-participation in the NMSP decreased. This area is characterized by a highly dense and deprived area composed of immigrants and women with lower mobility. We hypothesize that this spatial neighborhood confinement with a lower proportion of women aged for mammography screening may reduce the likelihood of being influenced by other women’s health behaviors. For Cluster 3, after adjusting for the deprivation and proportion of participation in screening unrelated to the national programme, the expected risk of non-participation in the NMSP decreased. Cluster 3 is the only cluster of census blocks with low participation of mammography screening without any mammography services inside.

Finally, our study emphasized that in the city center of Lyon, the level of deprivation did not explain the spatial distribution of participation in the NMSP observed in the crude analysis. Whereas the density of mammography services is very high, and the population is mostly less deprived (higher SES), the participation of breast cancer screening is very low. This is in contrast with the literature suggesting that women living in urban areas were more likely to have mammography screening. These results have been found previously in the province of Quebec by St-Jacques et al. called a rural–urban paradox [5]. They demonstrated that the lowest proportion of participants who used the nearest mammography service lived in the urban area of Montreal Island. They hypothesized that women might have selected a mammography screvice on criteria such as appointment times, convenient parking or accessibility by public transportation but not related to the distance. One previous study of Vallée et al. in 2010, located in Paris, demonstrated that in urban areas with a high practitioner density, inequalities in participation in preventive health care may not be directly associated with the spatial distribution of primary care providers, with the notable exception of people whose activity space is limited [41].

Further studies are needed to understand the factors and spillover effects in the city of Lyon that influence the participation of mammography sreening. To understand the urban–rural paradox, all dimensions of access, continuity, responsiveness and use of mammography services, could be analyzed in future studies to evaluate the gap between the breast cancer health care offered and that observed. Morover, the environmental pattern of our cluster of Lyon represents census blocks with favorable socioeconomic levels, accessibility of healthcare and transportation networks, but also higher residential mobility and dynamic populations. These factors were not favorable for participation in mammography screening. Finally, a previous study of Vogt (2014) demonstrated the importance of spillover effects in the analysis of regional variation in cancer screening utilization rates [12]. This means that individual preferences and knowledge regarding cancer screening are transmitted through personal contacts within social networks or neighborhoods. 

### 4.1. Limitations and Strengths

Regarding study strengths, the use of GIS and spatial analysis techniques has been shown to be useful to inform public policy and determine areas that warrant specific intervention. Moreover, this paper illustrates an approach to taking into account the combined effect between socioeconomic characteristics and others contextual risk factors to identify areas where combined conditions may have an impact on the participation in the NMSP. Further studies are needed to analyze the temporal variation of mammography screening, comparing these results with future results after interventions to see if the clusters persist.

Our study has some weaknesses related to missing integrated information in the classification of the census blocks. Firstly, we were not able to consider individual behaviors and attitudes towards participation of mammography sreening such as fear and cultural beliefs. Moreover, several French studies have been conducted with the aim of understanding the reasons for low mammography sreening participation among women from disadvantaged socioeconomic backgrounds [19]. These show that among vulnerable groups, health issues are considered less vital than everyday concerns such as food or shelter, and are therefore often not a priority [37]. However, an ecological indicator of deprivation could be a good proxy of behavior and individual factors highly related to the socioeconomic and material conditions [42]. Secondly, we could not therefore take into account factors related to access and healthcare supply, such as access using travel time and distance to mammography screening. Others additional risk factors hypothesized in the literature for which we unfortunately did not have at the individual-level include medical care consultation follow up and healthcare supply. Thirdly, we had limited information on screening unrelated to the NMSP other than data from the general medical care reimbursements, and these were limited to the municipality level except for the city of Lyon (census block level).

### 4.2. Usefulness of Spatial Analysis in Global Public Health

Small geographic areas with lower mammography screening participation rates may reflect gaps in screening efforts. In this context, spatial analysis using available data can provide important results to inform local health policies. This tool allows the early identification of areas in need of policymakers to focus the scope of prevention/intervention programs. Policy makers could then focus on an appropriate direction, depending on the particular area’s need, to achieve more efficient distribution of public resources. Moreover, spatial models related to screening could consider contextual factors or individual’s factors to improve our understanding of factors that could explain the spatial distribution of screening participation. To that end, low screening risk maps can be powerful tools for the facilitation of decision-making in public health, for surveillance purposes and with a view to a positive evolution of the screening coverage rate.

## 5. Conclusions

This study focused on detecting significant clusters at the census block level in Lyon MA for 2015 to 2016 at high risk of non-participation in the NMSP. In addition to the confirmation of clusters of higher non-participation in highly deprived areas, the spatial analyses offered useful information on the contribution of other risk factors to guide actors from the regional and local mammography screening programme on further interventions in specific areas of need. One cluster located in the city center remained unexplained. Future research could be focused on incorporating specific indicators related to access and distance to mammography screening to understand why the study found a cluster in the city center of Lyon where the number of mammography services is higher than the rest of the study setting. Moreover, general efforts should be put in place in all the cluster locations to explore and investigate potential and modifiable factors related to beliefs according to mammography screening.

## 6. List of Abbreviations

GIS: Geographic Information System, INSEE: National Institute of Statistics and Economic Studies, MA: Metropolitan area, NMSP: National Mammography Screening Programme

## Figures and Tables

**Table 1:** Description of the clusters of high risk of non-participation in the NMSP according to **contextual** characteristics of the census blocks in Lyon MA.

Number of Census Blocks within the Cluster	Cluster 1 (N = 2)	Cluster 2 (N = 37)	Cluster 3 (N = 27)	Cluster 4 (N = 71)
**Municipalities within the Cluster**	**Rillieux la pape**	**Decines (la soie)**	**Vénissieux**	**City of Lyon**
		**Villeurbanne**	**Saint Fons**	
		**Vaulx en velin**		
		**Bron**		
**Mammography screening**				
Prevalence of non-participation in NMSP (%)	56.5 ± 6.0	58.2 ± 7.4	57.1± 6.4	53.6 ± 6.4
Opportunistic screening part (%)	7.7 ± 0	8.1 ± 0.4	7.6 ± 0.3	9.9 ± 1.1
**Socioeconomic deprivation**				
Proportion of blue collar workers in the labor force (%)	18.9 ± 8.6	24.9 ± 11.2	29.5 ± 13.5	8.3 ± 5.6
Proportion of managers in the labor force (%)	25.5 ± 30.4	13.2 ± 11.9	5.2 ± 4.5	48.7 ± 18.2
People aged 15 years or older with a higher education degree (%)	68.5 ± 6.9	46.5 ±28.1	27.6 ± 16.7	96.6 ± 4.4
People aged 15 years or older who did not go beyond elementary education (%)	54.7 ± 15.5	72.4 ± 27.3	87.2 ± 38.4	29.2 ± 11.3
Proportion of unemployed people (%)	20.8 ± 13.4	25.1 ± 13.5	28.5 ± 17.8	19.8 ± 8.9
Proportion of foreigners in the total population (%)	35.4 ± 22.5	68.5 ± 31.5	73.8 ± 38.3	30.4 ± 13.5
Proportion of single parent family	15.2 ± 7.7	14.3 ± 6.4	16.8 ± 7.7	8.9 ± 3.9
Median income per consumption unit (mean ± SD)	19537 ± 1409	15328 ± 5404	13342 ± 3325	25959 ± 6119
**Social cohesion**				
Women aged 50 or more part (%)	15.3 ± 2.7	11.6 ± 2.3	11.6 ± 2.2	11.1 ± 5.5
